# Working conditions as risk factors for early exit from work—in a cohort of 2351 employees in Germany

**DOI:** 10.1007/s00420-020-01566-x

**Published:** 2020-09-15

**Authors:** Angelo d’Errico, Hermann Burr, Dagmar Pattloch, Norbert Kersten, Uwe Rose

**Affiliations:** 1 Department of Epidemiology, Local Health Unit TO 3, Turin, Italy; 2 Department of Work and Health, Federal Institute for Occupational Safety and Health (BAuA), Berlin, Germany

**Keywords:** Exit from work, Labour market participation, Sickness absence, Unemployment, Disability, Working conditions, Occupational exposures

## Abstract

**Objectives:**

We would assess the possible impact of a range of physical and psychosocial working conditions on early exit from paid employment (i.e., before retirement age) in a representative employee population in Germany.

**Methods:**

We analysed a cohort from the German Study on Mental Health at Work (S-MGA) with a baseline of 2351 employees in 2011/12, sampled randomly from the register of integrated employment biographies (IEB) at the Institute for Employment Research (IAB). Follow-up ended mid-2015. Early Exit comprised episodes of either pensioning, long-term sickness absence or unemployment ≥ 18 months. Total follow-up years were 8.422. Working conditions were partly assessed by the Copenhagen Psychosocial Questionnaire (COPSOQ). Through Cox regressions, associations of baseline working conditions with time to event of exit were estimated—adjusting for baseline age, gender, poverty, fixed-term contract and socioeconomic position.

**Results:**

In multiple regressions, awkward body postures (HR = 1.24; 95% CI = 1.07–1.44), heavy lifting (1.17; 1.00–1.37) and high work pace (1.41; 1.16–1.72) were associated with exit. The estimated attributable fraction of exit for being exposed to less than optimal work environment was 25%. Regarding specific exit routes, repetitive movements (1.25; 1.03–1.53) increased the risk for the long-term sickness absence; work pace (1.86; 1.22–2.86) and role clarity (0.55; 0.31–1.00) were associated to unemployment; and control over working time (0.72; 0.56–0.95) decreased the risk of the early retirement.

**Conclusions:**

Work environment seems to be important for subsequent early exit from work. Physical and psychosocial demands seem to be associated to exit to a stronger extent than resources at work.

## Introduction

An overall trend towards limiting access to pensioning before statutory pension age has taken place in many industrialized countries (Ebbinghaus and Hofäcker 2013), in order to improve the sustainability of the national social security systems, currently under pressure because of population ageing and increase in the age dependency ratio. In Germany, for example, participation in work has increased, but still many workers exit work before reaching statutory pension age (Buchholz et al. 2013). Depending on welfare state type and time period, early exit from work can take different paths, in relation to different national policies and economic cycle (Ebbinghaus and Hofäcker 2013). The predominant early exit routes from work are through retirement, i.e., disability pension and other types of early pensioning, and unemployment (Buchholz et al. 2013), but long-term sick leaves may be another alternative welfare programme accessed by older workers to definitively abandon the labour market until they reach the statutory pension age (Hultin et al. 2012; Labriola and Lund 2007; Pedersen et al. 2012; Wallman et al. 2009).

The choice of withdrawing earlier from the labour market is determined by several push and pull factors linked to societal, household, health-related and workplace characteristics, including mainly pension legislation, income, socioeconomic position (SEP), partnership status, health and work ability, and unfavourable working conditions (De Preter et al. 2013; Edge et al. 2017). The German context regarding early exit in the years 2011–15 was as follows: From the summer of 2014 employees aged ≥ 63 with a labour market seniority ≥ 45 years were eligible to early retirement at age 63, in practice this would apply to many skilled workers. Employees with employment biographies of 35 to < 45 years were entitled to retire with deductions in pension level (Deutsche Rentenversicherung [German pension insurance] 2020a). Employees were entitled to disability pension if no workability is left (Deutsche Rentenversicherung [German pension insurance] 2020b), and disability pension levels are low (672 € per month) (Deutsche Rentenversicherung [German pension insurance] 2016). In case of sickness absence, the employer continues to pay the wage for usually 6 weeks. Then, the health insurance begins to grant sick pay, which generally expires after 18 months. Sick employees are not safe from dismissal. The unemployment rate in Germany 2011–15 was moderate. It decreased from 7.9% (2011) to 7.1% (2015) (Statistisches Bundesamt [Federal Statistical Office of Germany] 2020). Unemployment compensation expires after 12 months followed by basic social benefits.

Regarding the impact of working conditions on early exit, only few studies have investigated global early exit as an outcome, i.e. not distinguishing between specific exit paths (Boot et al. 2014; de Boer et al. 2018; Lund and Borg 1999; Robroek et al. 2013a). The results of these studies seem to indicate that low job control and its sub-dimensions are the work factors most consistently associated with exit from work, whereas inconsistent findings have been reported for high physical and psychological demand.

In most studies, the role of working conditions on early exit from work was assessed by examining the association between exposure to work factors and only one or two exit routes. Among longitudinal studies conducted on the general working population, the most studied specific outcome was disability pension for all causes (Albertsen et al. 2007; Bödeker et al. 2008; Christensen et al. 2008; Clausen et al. 2014a, b; Hagen et al. 2002; Krause et al. 1997; Krokstad et al. 2002; Labriola et al. 2009; Lahelma et al. 2012; Laine et al. 2009; Lund and Csonka 2003; Lund et al. 2001, 2008; Mantyniemi et al. 2012; Robroek et al. 2013a; Ropponen et al. 2013; Samuelsson et al. 2013; Sinokki et al. 2010; Stattin and Jarvholm 2005; Tüchsen et al. 2010), followed by studies on long-term sickness absence (LTSA) (Andersen et al. 2016; Borritz et al. 2010; Burdorf and Jansen 2006; Christensen et al. 2007; Henderson et al. 2012; Lund et al. 2005, 2006; Lund and Lariola 2006; Melchior et al. 2003; Sterud 2014; Sundstrup et al. 2018a, b; Wang 2004). In contrast, fewer studies focussed on other types of early exit, such as early retirement (de Wind and van der Beek 2014; Friis et al. 2007; Lund et al. 2001; Lund and Villadsen 2005; Wind et al. 2017) or unemployment (Lund and Labriola 2006; Robroek et al. 2013b). Two studies collapsed early pension with disability pension into a global pension outcome (Robroek et al. 2013a; Siegrist et al. 2007).

All these types of early exit paths were mostly associated with high physical demands, and low job control, although the results in the literature were only partially consistent. Furthermore, most studies focussed only on a few work environment dimensions, such as physical demand and psychosocial factors related to the demand-control (DC) and the effort-reward imbalance (ERI) models (control, demand, strain, reward and social support from co-workers and supervisors) (Karasek and Theorell 1990; Siegrist 1996). However, other work factors, especially psychosocial ones, have been highlighted as possible risk factors for disability (Christensen et al. 2008; Clausen et al. 2014b; Emberland et al. 2017) and early retirement (Lund and Villadsen 2005; Thorsen et al. 2016; Breinegaard et al. 2017), as well as for LTSA (Lund et al. 2005; Sundstrup et al. 2018a; Borritz et al. 2010).

Therefore, a full picture of important risk factors for early exit from work is not possible to draw (Pohrt and Hasselhorn 2015). Moreover, most of these studies took place in smaller countries in Western and Northern Europe—i.e., the Netherlands and Scandinavia (Pohrt and Hasselhorn 2015), limiting their generalizability.

The aim of the present study was to assess the impact of a broad set of physical and psychosocial risk factors at work for early exit from paid employment before statutory retirement age, in a cohort of workers representative of the employed population in Germany.

## Materials and methods

### Population

**Fig. 1:**
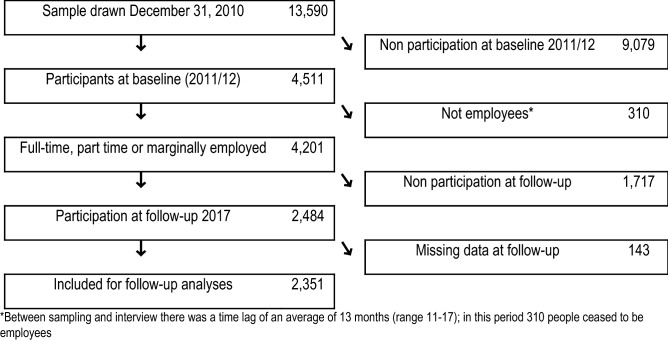
Flow diagram of participation in S-MGA’s 2011/12 baseline and the 2011/12–2015 cohort

We used data from the German Study on Mental Health at Work (S-MGA), which is a nation-wide representative employee cohort study with a baseline survey in 2011/12 and a follow-up in 2017 (Rose et al. 2017). At baseline, the target population consisted of all subjects employed in Germany on 31st December 2010, born in 1951–1980 (Rose et al. 2017). The study population was randomly sampled from the register of Integrated Employment Biographies (IEB) of the German Federal Employment Agency at the Institute for Employment Research (IAB). This register covers all employees in employment except civil servants, self-employed workers and freelancers. The analysed cohort comprised 2351 people employed at baseline (Fig. 1). At baseline, participation did not vary by gender, it was somewhat higher at older ages and higher among professionals, managers and semi-professionals (Table 1). Follow-up response was moderately associated with a number of working conditions: walking/standing and awkward body postures were associated with lower participation, whereas amount of work, control over working time and possibilities for development were associated with higher participation (Appendix Table 8). Subjects were followed up for early exit from paid employment until mid-2015, in order to allow the detection of episodes of long-term sickness absence or unemployment ≥ 18 months before the second data collection wave took place in mid-2017 (at the end of follow-up, the oldest workers were 63 years old). The number of total follow-up years was 8.422 (mean: 3.6 years).

**Table 1:** Participation in interviews at baseline, at follow-up and in the cohort by gender, age and occupational group

	Baseline response^a^, %	Follow-up response among baseline employees^b^, %	Cohort fraction of the drawn sample^c^, %
Sex			
Male	33	56	18
Female	33	56	19
Age			
55–60	39	52	20
49–54	35	57	20
43–48	33	56	18
37–42	32	58	18
31–36	27	55	15
SEP			
Professionals, managers	38	63	24
Semi-professionals	38	62	24
Skilled workers	32	54	17
Unskilled workers	29	49	14
Total	33	56	19

The table is based on published baseline and follow-up attrition analyses (Rose et al. 2017; Schiel et al. 2018)

^a^Fraction being interviewed at baseline (4511) of the drawn sample (13,590), see Fig. 1

^b^Fraction being interviewed at follow-up and with non-missing information (2351) of the employees interviewed at baseline (4201), see Fig. 1

^c^Fraction in the analysed cohort of the drawn sample (estimated by multiplying the fraction of the baseline response with the fraction of follow-up response among baseline employees)

### Variables

#### Outcome

Early exit was defined as having—before statutory pension age (65 years)—a first episode of:

- drawing a pension (early pension or disability pension);
- unemployment either lasting ≥ 18 months or < 1–17 months followed by transition into pension;
- sickness absence either lasting ≥ 18 months or < 1–17 months followed by transition into pension.

The 18 months cut-offs for both long-term sickness absence and long-term unemployment were established on the basis of the within-study-risk of exit > 75% at follow-up associated with duration of spells in the first half of the follow-up (Table not shown).

Information on early exit from paid employment was based on the questions in 2017 regarding each episode of employment and non-employment since 2011/12 (Borsch-Supan et al. 2013). Start—and possible end—of each episode was asked in year and month. During follow-up a total of 134 early exits occurred (6% of the cohort), of which 44 through long-term sickness absence (≥ 18 months or in combination with subsequent pension), 36 through unemployment (≥ 18 months or in combination with subsequent pension) and 54 through early or disability retirement.

### Independent variables

#### Physical demands

Exposure to four physical demands was collected through a 5-point Likert scales: ‘Never’ (0), ‘Up to a quarter of the time’ (1), ‘Up to half the time’ (2), ‘Up to three quarters of the time’ (3) and ‘More than three-quarters of the time, almost always’ (4).

The four physical exposures were as follows: Walking/standing, awkward work postures, lifting heavy loads and repetitive movements. Walking/standing was a scale assessed through questions on ‘Working standing’ and ‘Working while sitting’ (reverse scored). Cronbach’s α was 0.95; the inter-item correlation was 0.90. Awkward body postures was a scale based on the item ‘Bending, crouching, kneeling, lying or working with hands raised over shoulder height’. Lifting heavy loads was based on the following question: ‘Lifting and/or carrying heavy loads (women more than 10 kg/men more than 20 kg)’. Exposure to repetitive movements was based on the following question: ‘Repetitive movements, in the sense of repetitive physical activity’.

#### Psychosocial working conditions

The psychosocial working conditions mentioned below were measured by items from the German COPSOQ 1 inventory (Kristensen et al. 2005; Nübling et al. 2006) and had the following response options (and values for the scale): ‘Always’ (4), ‘Often’ (3), ‘Sometimes’ (2), ‘Seldom’ (1) and ‘Never/hardly ever’ (0)—apart from the quality of leadership scale (see below).

#### Domain: quantitative demands

Work pace was assessed through the single item (Kristensen et al. 2005; Nübling et al. 2006): “Do you have to work very fast”?

Amount of work was a scale calculated as the mean of four items from the scale “Amount of work” (Kristensen et al. 2005; Nübling et al. 2006): “Is your workload unevenly distributed so it piles up”? “How often do you not have time to complete all your work tasks”? “Do you get behind with your work”? “Do you have enough time for your work tasks”? (the last question was reversely coded). Cronbach’s α was 0.84; inter-item correlations were 0.47–0.68.

#### Domain: control

Influence at work (decision authority) was calculated as the mean of the following four items (Kristensen et al. 2005; Nübling et al. 2006): “Can you influence the amount of work assigned to you”? “Do you have any influence on what you do at work”? “Do you have a large degree of influence concerning your work”? “Do you have a say in choosing who you work with”? Cronbach’s α was 0.70; inter-item correlations were 0.31–0.43.

Control over working time was calculated as the mean of the three items (Kristensen et al. 2005; Nübling et al. 2006): “Can you decide when to take a break”? “Can you leave your work to have a chat with a colleague”? and “If you have some private business is it possible for you to leave your place of work for half an hour without special permission”? Cronbach’s α was 0.74; inter-item correlations were 0.47–0.49.

Possibilities for development (skill discretion) was computed as the mean of the three items (Kristensen et al. 2005; Nübling et al. 2006): “Is your work varied”? “Do you have the possibility of learning new things through your work”? and “Can you use your skills or expertise in your work”? Cronbach’s α was 0.69; the inter-item correlations were 0.39–0.46.

#### Domain: relations

Role clarity was calculated as the mean of the three items (Kristensen et al. 2005; Nübling et al. 2006): “Does your work have clear objectives”? “Do you know exactly which areas are your responsibility”? and “Do you know exactly what is expected of you at work”? Cronbach’s α was 0.69; the inter-item correlations were 0.36–0.51.

Quality of leadership was computed as the mean of the four items (Kristensen et al. 2005; Nübling et al. 2006): “To what extent would you say that your immediate superior…—makes sure that the individual member of staff has good development opportunities”? “—gives high priority to job satisfaction”? “—is good at work planning”? and “—is good at solving conflicts”? with the response options (and values for the scale): ‘To a very large extent’ (4), ‘To a large extent’ (3), ‘Somewhat’ (2), ‘To a small extent’ (1) and ‘To a very small extent’ (0). Cronbach’s α was 0.84; inter-item correlations were 0.52–0.64.

### Covariates

Information on gender and age were also collected through the interview.

Socioeconomic position was assessed by occupational skill level of each respondent inspired by Goldthorpe’s class theory and was treated as a categorical variable in the analyses (Goldthorpe 2000). Occupations were manually coded according to the International Standard Classification of Occupations (ISCO 08) and categorized into four groups: Unskilled workers (ISCO groups ‘8. Plant and machine operators, and assemblers’ and ‘9. Elementary occupations’), skilled workers (‘4. Clerical support workers’, ‘5. Service and sales workers’, ‘6. Skilled agricultural, forestry and fishery workers’ and ‘7. Craft and related trades workers’), semi-professionals (‘3. Technicians and associate professionals’) and managers/professionals (‘1. Managers’, ‘2. Professionals’) (Hagen 2015).

Fixed-term contract was based on the response option ‘—fixed term’ to the question “What is your current work contract?”.

Poverty was assessed through information on household income and number of adults and children in the household, using the official poverty definitions in Germany (Deutscher Bundestag [German Federal Parliament] 2011), based on a yearly minimum income of 7896 € for singles, and of 13,272 € for couples, with additional 4272 € per each child in the household.

### Data analysis

Through Cox regression models, associations of baseline working conditions with time to event of early exit from work during follow-up were estimated—adjusting for baseline age, gender, poverty, fixed-term contract and SEP (four categories: ‘Professionals’, ‘Semi-professionals’, ‘Skilled workers’ and ‘Unskilled workers’). Adjustment for age was performed by adding age to time to event (Chalise et al. 2016).

Signs of possible collinearity (Pearson’s correlation ≥ 0.25) were found (Vatcheva et al. 2016). Correlations above 0.40 were found between walking/standing, awkward body postures and lifting heavy loads; walking /standing and control over working time, work pace and amount of work; and influence and possibilities for development (Table 3). Correlations between 0.25 and 0.40 were found between control over working time and influence at work, lifting heavy loads and awkward body postures; and between quality of leadership and both amount of work and possibilities for development. These correlations had implications for the multiple regression analysis (see next paragraph).

In the main analysis predicting episodes of early exit from work, working conditions were in a first step entered separately in regression models adjusted for poverty, fixed-term contract and SEP [this analysis was repeated with design weights so as to see possible effects of attrition (Schiel et al. 2018)]. In a second step, each work environment factor was adjusted also for other work factors, but only for those belonging to other domains, and limiting the inclusion to only one work factor for each domain, in order to avoid collinearity issues (all correlations within domains above 0.20), except for the domain physical demands, where two factors were chosen. For this analysis, the factor showing the weakest correlation with work dimensions in other domains was selected, except for repetitive movements, which was also selected from the domain of physical demands because of its low correlation with other work factor in that domain. Therefore, the final set of work factors included awkward body postures and repetitive movements (domain: physical demands), work pace (domain: quantitative demands), influence (domain: control) and quality of leadership (domain: relations).

In a separate analysis, we investigated job strain as a predictor of early exit, first alone and afterwards adjusting for awkward body postures, repetitive movements and quality of leadership (we did not include work pace and influence at work as these two variables are part of the job strain measure). As job strain is a categorical variable, we treated the other work environment predictors as categorical variables, in order to better compare among each other the risks associated to the different exposures. In this analysis we also investigated if job strain, being a combination of high demands and low control, posed a risk above the sum of the risks of high demands and low control by calculating the Relative Excess Hazard Ratio due to Interaction (REHRI) (Rothman 2002). A significantly positive REHRI would indicate superadditivity (Andersson et al. 2005a; Rothman 2002).

In further analyses predicting the three early exit routes sickness absence, unemployment and pension, each exit route was investigated separately, and in each case censoring was applied to the other two exit routes. As in the main analysis, in a first step each individual work environment factor was entered without mutual adjustment. In a second step, the same selected work environment dimensions as in the main analysis were added to the regression model.

We did not stratify by gender, as gender did not interact with work environment dimensions. As both poverty and socioeconomic group interacted with gender (women being poor and/or being unskilled workers had the lowest risk for early exit), in all regressions models an interaction term between gender and poverty and/or being unskilled was added.

We also investigated if associations between working environment factors and early exit were non-linear by treating these as cubic terms. To illustrate possible non-linear associations, we treated working conditions in a special analysis as categorical, collapsing their scores in three exposure categories: low (0 to  < 1.5), medium (≥ 1.5 to  < 2.5) and high (≥ 2.5 to 4).

The risk of early exit attributable to exposure to those work environment dimensions significantly associated to exit in the mutually adjusted models—if any—was also estimated. These work environment dimensions were added together into an index ranging from 0 to 4, which was treated as a categorical variable with the following categories (and values): ‘Low (0 to < 1)’; ‘Below medium (1 to < 2)’; ‘Medium (2 to < 3)’ and ‘Above medium, high (3–4)’. The attributable fraction of early exit due to exposure to such factors was computed according to Miettinen’s method (Miettinen 1974). An attributable fraction can in our case be expressed as the fraction of events attributable to all risk factors found and can be illustrated graphically as the fraction of the area of bars over 1 of the total area of bars (Miettinen 1974).

There were no signs of violation to the proportional hazards assumption of the Cox approach.

Data were analysed by means of SPSS 20 using the COXREG command, except for the interaction analysis regarding job strain where the CSCOXREG command was used [this command yields a covariance matrix needed for calculating the variance of REHRI (Andersson et al. 2005b)].

## Results

The composition of the study population is shown in Table 2. Women constituted half of the sample. As can be seen, the scores of awkward body postures and heavy lifting had the lowest means, reflecting that these dimensions occurred to a lesser extent than the other work environment dimensions.

**Table 2:** Population

	Socioeconomic position (SEP)												Total (*N* = 2351)		
	Professionals, managers (*n* = 573)			Semi-professionals (*N* = 659)			Skilled workers (*N* = 846)			Unskilled workers (*N* = 273)					
	%	Mean	Std. dev	%	Mean	Std. dev	%	Mean	Std. dev	%	Mean	Std. dev	%	Mean	Std. dev
Age		46.4	7.8		46.4	7.4		47.0	7.7		47.8	7.3		46.8	7.6
Women	55			39			52			59			50		
Fixed term contract	6			3			5			9			5		
Poverty^a^	6			10			15			18			12		
Walk/stand^b^		1.05	1.04		1.38	1.24		2.34	1.51		2.87	1.35		1.82	1.47
Awkward body postures^b^		0.29	0.71		0.45	0.83		0.98	1.25		1.14	1.31		0.68	1.09
Lifting heavy loads		0.30	0.63		0.58	0.92		0.99	1.25		1.23	1.24		0.73	1.09
Repetitive movements^b^		1.13	1.41		1.34	1.47		1.41	1.46		1.62	1.53		1.34	1.46
Work pace^b^		2.59	0.92		2.69	0.91		2.69	0.99		2.71	1.09		2.67	0.97
Amount of work^b^		2.12	0.88		1.93	0.89		1.63	0.91		1.32	0.85		1.80	0.92
Influence^b^		2.11	0.77		1.67	0.90		1.58	0.99		1.25	0.97		1.70	0.95
Control over working time^b^		2.60	1.18		2.28	1.16		1.96	1.13		1.60	1.14		2.16	1.20
Possibilities for development^b^		3.18	0.59		2.96	0.65		2.70	0.78		2.05	1.00		2.82	0.81
Role clarity^b^		3.29	0.61		3.32	0.56		3.30	0.55		3.27	0.57		3.30	0.57
Quality of leadership^b^		2.26	0.85		2.20	0.92		2.32	0.93		2.22	0.95		2.26	0.91

^a^Official German definition based on household income and number of adults and children in the household (Deutscher Bundestag [German Federal Parliament] 2011).

^b^Scale ranging from 0 (low) to 4 (high) expressing the values of the underlying items having five response categories (see Sect. “Materials and Methods”)

Most working conditions were correlated to a greater extent to other working conditions belonging to the same domain than to variables from other domains (Table 3). Control over working time from the Control Domain constitutes an exception, as it was correlated also to a number of physical demands. The highest correlations were found within the physical demand domain. Also, physical demands were negatively correlated to high SEP, whereas all working conditions in the control domain and also amount of work were positively correlated to high SEP (see also Table 2). In a special analysis, we tested if the job strain category (job strain versus all other categories) was correlated to awkward body postures, repetitive movements and quality of leadership (all variables we aimed to control for in a special analysis described below; see Table 4). Here, the highest association to job strain was found with quality of leadership (− 0.21), the second highest with repetitive movements (0.10) and the lowest with demanding body postures (− 0.01) (Table not shown). Job strain was not associated to SEP (− 0.07).

**Table 3:** Correlations between the independent variables

		Covariates		Physical demands				Quantitative demands		Control			Relations	
		Age^a^	SEP^a^	Walk/stand	Awkward body postures	Lifting heavy loads	Repetitive movements	Work pace	Amount of work	Influence	Control over working time	Possibilities for development	Role clarity	Quality of leadership
Covariates	Gender (M = 1, W = 2)	0.03	0.03	− 0.03	− 0.08	− 0.04	0.07	0.10	− 0.02	− 0.14	− 0.021	− 0.05	0.05	0.04
	Age^a^	1	− 0.05	− 0.02	− 0.08	− 0.09	0.01	− 0.09	− 0.08	− 0.03	− 0.05	− 0.05	0.07	− 0.02
	SEP^a^		1	− 0.43	− 0.29	− 0.30	− 0.09	− 0.04	0.28	0.27	0.27	0.39	0.01	0.02
Physical demands	Walk/stand				0.54	0.55	− 0.13	0.06	− 0.15	− 0.08	− 0.43	− 0.15	0.03	0.04
	Awkward body postures				1	0.60	0.01	0.11	− 0.01	− 0.03	− 0.28	− 0.04	0.05	0.04
	Lifting heavy loads					1	0.04	0.17	0.04	− 0.07	− 0.30	0.03	0.03	0.01
	Repetitive movements						1	0.15	0.13	− 0.17	− 0.05	− 0.14	− 0.02	− 0.07
Quantitative demands	Work pace							1	0.40	− 0.12	− 0.18	− 0.02	0.01	− 0.10
	Amount of work								1	− 0.05	− 0.03	0.14	− 0.14	− 0.25
Control	Influence									1	0.37	0.41	0.12	0.18
	Control over working time										1	0.22	0.03	0.07
	Possibilities for development											1	0.23	0.26
Relations	Role clarity												1	0.21

^a^Treated as a continous variable. High levels represent high age, and high SEP

**Table 4:** Associations between baseline work environment dimensions and 134 events 2011/12–2015 of early exit from work^a^ among 2351 employees aged 31–60 years in Germany

Domain	Work environment dimension	Model 1. Work environment dimensions not mutually adjusted^b^		Model 2. Work environment dimensions mutually adjusted^c^	
		HR^b^	95% CI	HR^c^	95% CI
Physical demands	Walking, standing^d^	1.02	0.90–1.15	1.02	0–90–1.16
	Awkward body postures^d^	**1.25**	**1.08–1.46**	**1.24**	**1.07–1.44**
	Lifting heavy loads^d^	**1.18**	**1.06–1.31**	**1.17**	**1.00–1.37**
	Repetitive movements^d^	**1.17**	**1.04–1.30**	1.10	0.98–1.23
Quantitative demands	Work pace^d^	**1.53**	**1.26–1.85**	**1.41**	**1.16–1.72**
	Amount of work^d^	**1.34**	**1.10–1.63**	1.20	0.98–1.46
Control	Influence at work^d^	**0.80**	**0.67–0.96**	0.85	0.71–1.02
	Control over working time^d^	**0.82**	**0.70–0.95**	0.89	0.75–1.04
	Possibilities for development^d^	0.91	0.73–1.14	1.00	0.79–1.26
Relations	Role clarity^d^	1.11	0.80–1.55	1.09	0.78–1.52
	Quality of leadership^d^	**0.82**	**0.69–0.99**	0.86	0.71–1.03

Multiple cox regression, hazard ratios (HR). Bold numbers indicate sigificant HR's.

^a^Events of either sickness absence (≥ 18 months or combined with subsequent pension), unemployment (≥ 18 months or combined with subsequent pension) or pension in a 3.6-year follow-up

^b^Adjusted for gender, poverty, fixed-term contract, SEP (4 categories, see Sect. “Materials and methods”) and an interaction term (for gender and poverty and/or low SEP). Age was controlled for by adding age to time to event (Chalise et al. 2016)

^c^Adjusted for gender, poverty, fixed-term contract, socioeconomic statu, an interaction term (for gender and poverty and/or low SEP) and the following work environment dimensions from domains other than the domain to which the dimension belongs: Awkward body postures and repetitive movements (domain: physical demands), work pace (domain: quantitative demands), influence (domain: control) and quality of leadership (domain: relations). Age was controlled for by adding age to time to event (Chalise et al. 2016)

^d^Range of the variable: 0 low and 4 high expressing all values of the underlying items having five response categories (see Sect. “Materials and methods”)

Table 4 presents Hazard Ratios of early exit associated with 1-point increase (or decrease for the reversed scales) in the scores of each exposure, from regression models adjusted for sociodemographic covariates only (model 1), and further adjusted for the other work factors (models 2a, 2b and 3).

In the models adjusted for age, gender, poverty, fixed-term contract and SEP, 8 of 11 work environment dimensions were associated to subsequent early exit (Table 4, model 1), with the only exceptions of walking/standing, possibilities for development and role clarity (*p* = 0.808, 0.410 and 0.524, respectively). Physical and psychosocial demands increased the risk of early exit (awkward body postures, heavy lifting, repetitive movements, work pace and amount of work), whereas psychosocial resources lowered it (influence at work, control over working hours and quality of leadership). Work pace was the work factor showing the strongest association with early exit, with an approximately 50% higher risk for an increase of one point in the exposure score. In an analysis weighted so as to adjust for cohort attrition (see Table 1) (Schiel et al. 2018), results were unchanged (Appendix Table 9).

Considering physical and psychosocial factors together in a single model (model 2, Table 4), awkward body postures, heavy lifting and high work pace remained associated with a higher risk of early exit—together with repetitive movements, however, only marginally significantly, while influence at work and quality of leadership showed a marginally significant lower risk (model 2, Table 4). Taking into account the whole range of scores for the three significant risk factors, the results indicate that the risk for early exit was 70% higher for the highest level of exposure to heavy lifting versus the lowest level, it doubled for the highest level of awkward body postures versus the lowest, and that it increased by more than 180% between the highest and lowest level of work pace.

In a separate analysis, job strain more than doubled the risk of early exit in the analysis unadjusted for other work environment dimensions, but it attenuated by one-third and lost significance when adjusting for the work factors found associated in the fully adjusted model, i.e. awkward body postures, repetitive movements and quality of leadership (Table 5). The risk for job strain was higher than the sum of those estimated for low control (passive work) and high demands (active work); the REHRI was positive, i.e., significantly above 0, and amounted to 1.31 (95% CI: 0.27–2.34) in the unadjusted model and 1.05 (0.20–1.90) in the adjusted model.

**Table 5:** Associations between baseline job strain status and 134 events 2011/12–2015 of early exit^a^ from work among 2351 employees aged 31–60 years in Germany

	*N*	Observed cumulative incidence of early exit	Model 1. Job strain					Model 2. Job strain additionally controlled for awkward body postures, repetitive movements and quality of leadership					
		% (*n*)	*p*^b^	HR^c^	95% CI	REHRI^c,d^	95% CI	*p*^b^		HR^e^	95% CI	REHRI^c,d^	95% CI
Job strain			**0.006**					**0.038**					
No strain (low demands, high infl.)	348	5 (18)		1						1			
Passive (low demands, low influence)	863	7 (60)		1.02	0.59–1.78					0.90	0.51–1.58		
Active (high demands, high influence)	880	4 (34)		0.82	0.46–1.48					0.79	0.44–1.42		
Strain (high demands, low influence)	260	8 (22)		**2.15**	**1.13–4.11**	**1.31**	**0.27–2.34**			1.74	0.90–3.38	**1.05**	**0.20–1.90**

Multiple cox regression. Hazad ratios (HR). Bold numbers indicate sigificant HR's

^a^Events of either sickness absence (≥ 18 months or combined with subsequent pension), unemployment (≥ 18 months or combined with subsequent pension) or pension in a 3.6-year follow-up.

^b^This *p* value denotes—in the cox regression—to what extent the categorical job strain variable is associated with early exit from work

^c^Adjusted for gender, poverty, fixed-term contract, SEP (4 categories, see Sect. “Materials and methods”) and an interaction term (for gender and poverty and/or low SEP). Age was controlled for by adding age to time to event (Chalise et al. 2016).

^d^Relative excess hazard ratio due to interaction. A positive value above 0 expresses superadditivity, i.e., the overserved combined effect of low control and high demands is above the additive effect of these two factors (Andersson et al. 2005a; Rothman 2002).

^e^Adjusted for gender, poverty, fixed-term contract, SEP, an interaction term (for gender and poverty and/or low SEP), awkward body postures, repetitive movements and quality of leadership. Age was controlled for by adding age to time to event (Chalise et al. 2016).

In three further analyses (Table 6), each of the three early exit routes was investigated, also through a model including sociodemographic covariates only and one with mutual control for other work factors, as for the main analysis. Regarding LTSA, lifting heavy loads, repetitive movements, work pace and amount of work increased the risk, whereas quality of leadership decreased it (Table 6, first columns), but when taking other work environment dimensions into account, only repetitive movements remained associated. Regarding the unemployment route, in the fully adjusted model only work pace increased the risk, whereas role clarity decreased it (Table 6, middle columns). Last, control over working time was found to decrease the risk of early retirement, without any other work factor significantly associated (Table 6, last columns).

**Table 6:** Associations between selected baseline work environment dimensions and events^a^ 2011/12–2015 of sickness absence, unemployment or pension among 2351 employees aged 31–60 years in Germany

Domain	Work environm factor	Sickness absence^b^ (44 events)				Unemployment^b^ (36 events)				Pension^b^ (54 events)			
		Model 1^c^		Model 2^d^		Model 1^c^		Model 2^d^		Model 1^c^		Model 2^d^	
		HR^c^	95% CI	HR^d^	95% CI	HR^c^	95% CI	HR^d^	95% CI	HR^c^	95% CI	HR^d^	95% CI
Phys. dem	Walking, standing^e^	1.24	0.98–1.59	0.98	0.79–1.21	0.96	0.76–1.22	0.93	0.72–1.19	1.08	0.89–1.31	1.10	0.90–1.34
	Awkward body postures^e^	1.27	1.00–1.61	1.23	0.97–1.55	1.28	0.96–1.70	1.24	0.93–1.66	1.22	0.95–1.56	1.23	0.95–1.59
	Lifting heavy loads^e^	**1.29**	**1.02–1.64**	1.18	0.93–1.51	1.18	0.87–1.59	1.10	0.81–1.49	1.15	0.88–1.50	1.13	0.86–1.50
	Repetitive movements^e^	**1.33**	**1.10–1.61**	**1.25**	**1.03–1.53**	1.10	0.89–1.37	1.02	0.82–1.27	1.26	0.94–2.68	1.04	0.86–1.25
Quant. dem	Work pace^e^	**1.54**	**1.10–2.16**	1.33	0.95–1.85	**2.00**	**1.31–3.04**	**1.86**	**1.22–2.86**	1.22	0.91–1.63	1.21	0.90–1.62
	Amount of work^e^	**1.53**	**1.10–2.11**	1.26	0.90–1.76	1.43	0.99–2.07	1.27	0.87–1.86	1.11	0.80–1.53	1.05	0.76–1.46
Control	Influence at work^e^	0.72	0.51–1.00	0.84	0.60–1.18	0.80	0.55–1.14	0.89	0.62–1.28	0.86	0.65–1.13	0.83	0.63–1.10
	Control over working time^e^	0.81	0.62–1.05	0.93	0.71–1.23	0.96	0.72–1.29	1.14	0.83–1.55	**0.73**	**0.57–0.94**	**0.72**	**0.56–0.95**
	Possibilities for development^e^	0.85	0.59–1.23	1.00	0.69–1.47	1.11	0.70–1.75	1.24	0.79–1.97	0.88	0.61–1.27	0.85	0.58–1.25
Relations	Role clarity^e^	1.67	0.92–3.03	1.67	0.92–3.03	0.57	0.32–1.03	**0.55**	**0.31–1.00**	1.37	0.80–23.37	1.35	0.78–2.36
	Quality of leadership^e^	**0.72**	**0.53–0.97**	0.79	0.58–1.08	0.73	0.51–1.03	0.77	0.54–1.09	1.04	0.76–1.40	1.04	0.76–1.44

Cox regression, hazard ratios (HR). Bold numbers indicate sigificant HR's

^a^Events in a 3.6-year follow-up

^b^≥ 18 months or combined with subsequent pension

^c^Adjusted for gender, poverty, fixed-term contract, SEP (4 categories, see method section) and an interaction term (for gender and poverty and/or low SEP). Age was controlled for by adding age to time to event (Chalise et al. 2016). Work environment dimensions not mutually adjusted

^d^Adjusted for gender, poverty, fixed-term contract, SEP (4 categories, see Sect. “Materials and methods”) and an interaction term (for gender and poverty and/or low SEP) and the following work environment dimensions from domains other than the domain to which the dimension belongs: Awkward body postures and repetitive movements (domain: physical demands), work pace (domain: quantitative demands), influence (domain: control) and quality of leadership (domain: relations). Age was controlled for by adding age to time to event (Chalise et al. 2016)

^e^Range of the variable: 0 low and 4 high expressing all values of the underlying items having five response categories (see Sect. “Materials and methods”)

There were some signs of non-linear associations between the work environment factors and early exit regarding three working conditions, namely awkward body postures, work pace and leadership quality. In these three cases cubic terms of these work environment factors predicted early exit better than linear terms (regarding awkward body postures *p* for the cubic term was 0.000274 whereas *p* for the linear term was 0.003; regarding work pace the corresponding *p* values were 0.000006 versus 0.0002; regarding leadership quality 0.004 versus 0.035). This is illustrated when treating working conditions as categorical variables (Appendix Table 10). Regarding these three working conditions, risks for early exit were only elevated (i) when reporting awkward body postures at least ‘¾ of working hours’; (ii) on average reporting work pace items at least ‘sometimes’ (and the risk did not increase with higher levels) and iii) on average reporting quality of leadership items ‘To a very small extent’.

**Fig. 2:**
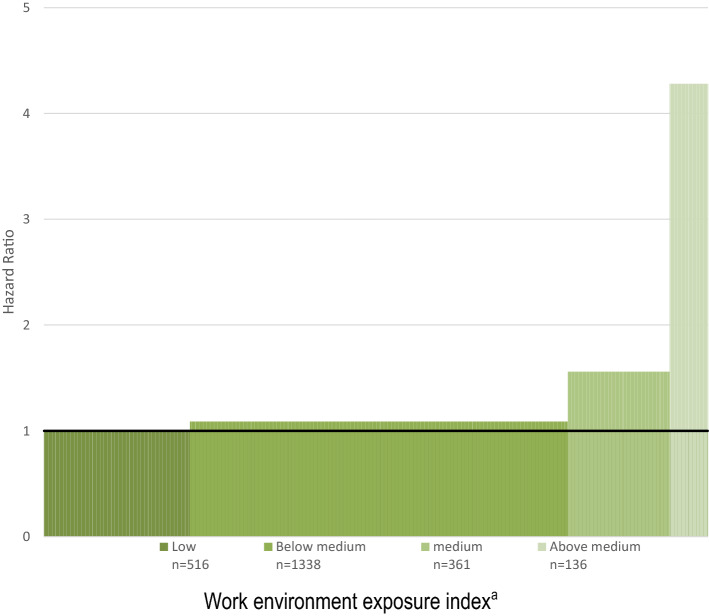
Risk of early exit from work 2011/12–2015^a^ by levels of a work environment exposure index^b^ among 2351 employees aged 31–60 years in Germany. Multiple cox regression. Hazard ratios (HR’s). The hight of each bar represents HR’s of each level of the work environment exposure index. The width of each bar represents its prevalence in the population (n’s). The total area of the bars represents all exits; the area of the bars above HR = 1 represents number of exits attributable to the work environment exposure index. The fraction of the area attributable to less than optimal scores of the work environment exposure index of the total area is 25% (Miettinen 1974). See also Table 7. ^a^Adjusted for gender, poverty, fixed term contract, SEP (4 categories, see Sect. “Materials and methods”) and an interaction term (for gender and poverty and/or low SEP). ^b^An index combining the work environment dimensions awkward body postures, heavy lifting and work pace (see Table 7)

The estimated attributable fraction of early exit for being exposed to less than optimal work environment was 25%. An illustration of this fraction is depicted in Fig. 2. In the figure, the area of the bars above 1 represents those exits attributable to levels of a work environment index with less than optimal scores. This area takes up 25% of the total area of these bars (Miettinen 1974) (Fig. 2). A less than optimal work environment consisted here of a mean value of at least 1 of an index going from 0 to 4 computed as the mean of the scores of the dimensions awkward body postures, heavy lifting and work pace (each also scoring from 0 to 4). The index was collapsed into four categories: ‘Low (0 to < 1)’; ‘Below medium (1 to < 2)’; ‘Medium (2 to < 3)’ and ‘Above medium, high (3–4)’. An optimal work environment was defined as an average score of < 1 on this index, reflecting the response category ‘Never’ to awkward body postures and heavy lifting and ‘Never/hardly ever’ to work pace, which was experienced by 22% of the population. This categorical measure was significantly associated to early exit (*p* = 0.018; Table 7; Fig. 2).

**Table 7:** Associations between a baseline work environment exposure index^a^ and 134 events 2011/12–2015 of early exit from work^b^ among 2351 employees aged 31 to 60 years in Germany

	*N* (Fraction of total, %)	Observed cumulative incidence of early exit, %	*p*^c^	HR^d^	95% CI
Work environment exposure index^a^			0.018		
Low (0 to < 1)^e^	516 (22)	4		1	
Below medium (1 to < 2)^f^	1338 (57)	5		1.09	0.48–2.50
Medium (2 to < 3)^g^	361 (15)	9		1.56	0.59–4.11
Above medium, high (3–4)^h^	136 (6)	8		**4.28**	**1.51–12.16**

Multiple cox regression, hazard ratios (HR). Bold numbers indicate sigificant HR's. See also Fig. 2

^a^An index being the mean of the following three work environment dimensions (each ranging from 0–4): awkward body postures, heavy lifting and work pace

^b^Events of either sickness absence (≥ 18 months or combined with subsequent pension), unemployment (≥ 18 months or combined with subsequent pension) or pension in a 3.6-year follow-up

^c^This *p* value denotes—in the cox regression—to what extent this categorical variable is associated with early exit from work

^d^Adjusted for gender, poverty, fixed term contract, SEP (4 categories, see Sect. "Materials and methods") and an interaction term (for gender and poverty and/or low SEP). Age was controlled for by adding age to time to event (Chalise et al. 2016)

^e^Reflecting response categories on individual items such as ‘Never’ to questions on awkward body postures and heavy lifting and ‘”Never/hardly ever” to questions on work pace

^f^Reflecting response categories on individual items such as ‘Up to a quarter of the time’ to questions on awkward body postures and heavy lifting and ‘”Seldom” to questions on work pace

^g^Reflecting response categories on individual items such as ‘Up to half of the time’ to questions on awkward body postures and heavy lifting and ‘Sometimes” to questions on work pace

^h^Reflecting response categories on individual items such as ‘Up to three quarters of the time’ or ‘More than three-quarters of the time’ to questions on awkward body postures and heavy lifting and ‘Often’ or ‘Always” to questions on work pace

## Discussion

The present study indicates that work demands, such as awkward body postures, lifting heavy loads and work pace, are associated with an increased risk of early exit from work, whereas resources at work, such as influence and quality of leadership, might be associated, although with a lower strength, with a decreased risk of early exit. In fact, our analyses suggest that a quarter of early exits are attributable to awkward body postures, lifting heavy loads and work pace. The study also indicates some non-linear associations; the risk of early exit for exposure to awkward body postures was only elevated at very high levels, the risk associated with high work pace was elevated already at relatively low levels (and did not increase with further increasing levels), while the risk for exposure to low quality of leadership was elevated at only very low levels.

An increased risk of early exit associated with physical work demands clearly emerges, possibly attributable to exposure to awkward postures or heavy lifting, although, because of their high intercorrelation, it was not possible to enter in the model simultaneously these two variables to determine their individual effect. Another important risk factor for early exit appears to be that of psychological demands, captured by the variables work pace and amount of work, but also for these dimensions their strong correlation limited the possibility to estimate reliably their independent effect in a multiple regression model. Although work pace may be an indicator of physical demand, it showed only a mild correlation with physical factors (Pearson correlation 0.06–0.17), allowing to assess the effect of exposure to high psychological demand controlling for physical demand and other work factors (adjustment for two physical factors and two psychosocial factors decreased the risk by one fifth). Another possible predictor for early exit might be job control, although influence at work was only marginally associated to early exit in the mutually adjusted model; maybe its effect is too small to be detected in this population (143 premature exits out of 2351 employees).

This study also showed that some physical and psychosocial work dimensions proposed in the literature partly overlap. High correlations were observed especially within work domains, for example, between work pace and amount of work (domain of quantitative demand), between control over working time and influence at work (domain of control), or among different factors in the domain of physical demand (awkward body postures, heavy lifting and walking/standing) (Table 3). From a theoretical point of view, there is a lack of understanding of the interdependence—and uniqueness of—specific working condition dimensions. Statistically this problem shows itself through two insufficient solutions: in multiple regressions, it poses problems to do mutual adjusted analyses when entering all working conditions into the same model, as also intercorrelations as low as 0.25 would lead to multicollinearity, making interpretations of risks impossible (Vatcheva et al. 2016); to solve this problem by constructing metascales, such as demands and resources, poses other problems, because of possible differential subscale effects (Burr and d’Errico 2018). In the present paper, we therefore did mutually adjusted regression models where we only controlled for a limited number of working conditions (Tables 4, 5).

Also, the study seems to indicate an association between the job strain construct and early exit. We found that job strain ceased to predict early exit when controlled for physical demands and quality of leadership. One could argue that these covariates in part contribute to high demands and low control; thus, adjustment for these factors might represent an overadjustment. Further, the study indicated that demands and control interact, i.e., job strain poses a risk for early exit over and above what one could expect when considering the respective risks of demands and control. More well-powered studies should look more at possible interactions among working conditions on early exit.

The results on specific early exit routes seem to indicate that the working environment has somewhat stronger associations to the sickness absence route than to those of unemployment and pensioning. Only work pace and role clarity were associated with the unemployment route, and only control over working time decreased the risk of taking the pension route. However, due to the low number of events, the results on single exit routes should be interpreted with caution. The work environment risk factors found—lifting heavy loads, repetitive movements, work pace and amount of work—have been found to be associated to subsequent poor mental health and/or musculoskeletal complaints leading to sickness absence (da Costa and Vieira 2010; Theorell et al. 2015).

### Strengths

This study of employees with a broad age range and examining several work environment dimensions is the first of its kind in Germany. A major strength of this study is that it used validated instruments, such as COPSOQ (Nübling et al. 2006; Pejtersen et al. 2010), and the employment history tool (Borsch-Supan et al. 2013). Second, the study is relatively large, including 2351 subjects. Third, the adjustment for several societal and household covariates, in particular for SEP and income, is expected to have reduced the possibility that the observed associations have been confounded by other subjects’ characteristics. We did not consider a control for SEP as an overadjustment, as SEP is expected to have a major independent impact on early exit from work (Schuring et al. 2013; Visser et al. 2016). Also, the control for other physical and psychosocial exposures in the fully adjusted model on early exit from work allows excluding relevant distortions in the risk estimates due to confounding by other work factors, in contrast to most other studies on the subject. Fourth, this analysis has dealt with more exit routes out of work apart from pensioning, namely sickness absence and unemployment, which are more difficult to operationalize as these states could be recurrent. People taking these routes would in many cases only in retrospect see them as exit routes. We have used two quite stringent criteria to define the exit routes sickness absence and unemployment. Either sickness absence or unemployment disregarding duration had to be followed by pension, or sickness absence or unemployment had to last at least 18 months. This length was chosen, because it was associated to a high risk (< 75%) of later early exit (it is a pure coincidence that we found the same duration cut point for both these routes). Future studies with access to labour market data covering a longer period may enable a definition of early exit routes in a more refined way.

### Limitations

The strengths of this study need to be balanced against its weaknesses.

First, this study is observational, where selection bias has to be considered, also in the light of the low participation in the cohort. Based on comparisons with the study’s sampling frame, differences in participation at baseline by gender and age were limited (Table 1), whereas they were greater between SEP strata, with a response fraction almost 10% lower among unskilled workers, compared to professionals, managers and semi-professionals. Similar differences by SEP were also observed for participation at follow-up. Several other researchers have reported lower participation in surveys and epidemiological studies among subjects in more disadvantaged social positions (Cifuentes et al. 2008; Demarest et al. 2013; Goldberg et al. 2001; Goyder et al. 2002; Lissner et al. 2003), for reasons which are still not well understood. Differences in attrition by level of exposure to the different work factors were of magnitude similar to those observed by socioeconomic position (Appendix Table 8) and appeared, at least in part, explained by their association with socioeconomic position, as high levels of walking/standing and awkward body postures were found in lower social class, whereas high levels of amount of work, control over working time and possibilities of development were found in higher social class (Table 3). SEP differences in attrition are not expected to have caused a substantial distortion of the associations away from true effects, considering that differences were relatively small (maximum difference in response rate at follow-up: 13% points between subjects exposed to low or high possibilities for development), and that all analyses were adjusted for socioeconomic position. An analysis with design weights so as to control for attrition did not change the results (Appendix Table 9).

Second, all physical demand dimensions were only measured with one to two items. This might lead to some measurement error regarding these variables and to a consequent non-differential misclassification of the exposures, which in turn would produce an underestimation of their associations with early exit.

Third, the study did not consider social support, as we had concerns regarding the validity of the available COPSOQ 1 question in the S-MGA (Burr et al. 2019). Social support from supervisors has been shown to be strongly correlated to quality of leadership (Burr et al. 2019), which we included as possible risk factor in the present study. In contrast, we could not assess possible effects of social support from colleagues.

Fourth, as the study was based on self-reports, it cannot be ruled out that people with poor health have exaggerated physical or psychosocial demands and underestimated psychosocial resources, which could have led to an overestimation of the associations, if subjects with poorer health, as expected, were more likely to exit from work.

Fifth, we treated work environment variables as continuous variables assuming a linear association. In a sensitivity analysis we—as mentioned above—did only find few signs of non-linearity by treating working conditions as cubic terms.

Sixth, we assessed SEP through occupational social class. This approach overlooks important aspects of the complexity of SEP related to the household and to lifetime biographies. Unfortunately, the study did not entail such data.

Last, exposure to workplace factors was assessed only at baseline, but it could have changed during follow-up, possibly causing a non-differential misclassification of the exposure and an attenuation of the associated risk estimates.

### Comparison with earlier studies

The comparability of our results with other studies on early exit as a global measure is limited by differences regarding work dimensions and data analyses (Boot et al. 2014; de Boer et al. 2018; Lund and Borg 1999; Robroek et al. 2013a). One study on Danish employees found that possibilities for development lowered risk of early exit in both genders, and—among women—also decision authority and medium level of social support. A lack of control for exposure to physical or psychosocial demands might overestimate the role of psychosocial resources (Lund and Borg 1999). Two Dutch studies also include physical demands, but their results were stratified by chronic disease status (Boot et al. 2014; de Boer et al. 2018). In the first one, the risk of early exit increased with physical demands and decreased with psychosocial resources, but only among subjects affected by chronic diseases, and increased with high psychosocial demands in the overall sample (Boot et al. 2014). In the other study, only time pressure increased the risk of early exit at 1-year follow-up, this was also the case with emotional demands at 2-year follow-up, in both cases only among workers with chronic diseases, whereas physical demands was not associated (de Boer et al. 2018). A European study based on SHARE—examining the association of early exit with physical and psychosocial demands, job control and rewards—found an increased risk of early exit for exposure to low job control and low rewards, but no association with physical demand and high time pressure (Robroek et al. 2013a); however, adjustment for health status may have led to an underestimation of the effect of work factors in this study, due to the possible mediating role of health.

Also, the finding of a positive association between physical demands and early exit in our study appears consistent with the results of a recent Danish study, which did not consider a single global exit route but four routes separately, and found significant associations between exposure to high physical demands and exit from work through disability pensions, early retirement, and LTSA, whereas the increase in risk was only marginally significant for unemployment (Sundstrup et al. 2018a).

Among studies investigating only one or two exit routes from paid employment, exposure to physical factors at work has been quite consistently associated with an increased risk of exit through disability retirement (Albertsen et al. 2007; Bödeker et al. 2008; Emberland et al. 2017; Karpansalo et al. 2002; Krause et al. 1997; Krokstad et al. 2002; Labriola et al. 2009; Lahelma et al. 2012; Lund and Csonka 2003; Pohrt and Hasselhorn 2015; Stattin and Jarvholm 2005) and early retirement (Friis et al. 2007; Lund et al. 2001, 2005).

Exposure to physical workload has also been found to increase the risk of unemployment (Borg and Burr 1997; Lund et al. 2001; Robroek et al. 2013a), although these studies mainly examined shorter periods of unemployment, which could not be considered a definitive exit from paid employment.

Physical demand has been found associated also with LTSA in several studies (Andersen et al. 2016; Burdorf and Jansen 2006; Christensen et al. 2007; Lund et al. 2006; Lund and Labriola 2006; Melchior et al. 2005; Sterud 2014) with higher risks generally found among blue-collar workers. However, for this outcome the comparability with our results is expected to be limited, because the LTSA definition used in our study was of much longer duration than that employed in the referenced studies, which mainly adopted a cut-off of few weeks. In these studies, physical risk factors for early exit included mainly physical demand or similar indicators of physical workload, with only few reporting associations with specific exposures, such as repetitive movements (Labriola et al. 2009), bending of the back or neck (Albertsen et al. 2007; Lund et al. 2001), or working in awkward postures (Albertsen et al. 2007; Karpansalo et al. 2002; Krause et al. 1997; Labriola et al. 2009; Lund and Csonka 2003).

Psychosocial factors at work have also been found associated in several studies with an increased risk of disability pensions, although a recent review on 39 studies concluded that there is only moderate evidence of an association for low job control and job strain, and limited evidence for other psychosocial dimensions, such as job demands, effort-reward imbalance, low social support and repetitive work (Knardahl et al. 2017).

Non-disability retirement also appears to increase mainly by low job control or its sub-dimensions (Blekesaune and Solem 2016; de Wind and van der Beek 2014; Lund and Villadsen 2005; Robroek et al. 2013a; Thorsen et al. 2016), but other factors, such as low role clarity, low reward, low organizational justice and low leadership or management quality, have been reported among risk factors (Breinegaard et al. 2017; Thorsen et al. 2016). A recent review on the relationship between exposure to psychosocial factors at work and early retirement concluded that there is sufficient evidence that high job control and high social support are associated with later retirement, but not for job demands, organizational justice, effort-reward imbalance or other psychosocial work factors (Browne et al. 2018).

Unemployment was consistently associated with low control and its sub-dimensions in the few available studies (Lund et al. 2001; Lund and Labriola 2006; Robroek et al. 2013a), whereas various psychosocial factors have been found to increase the risk of LTSA, but results appear inconsistent among studies; nonetheless, low control or its sub-dimensions have been repeatedly associated with LTSA (Henderson et al. 2012; Lund et al. 2005; Melchior et al. 2005). Also, one study reported an increased risk of LTSA for exposure to high strain (Wang et al. 2004), while another one also for exposure to conflict, rewards, quality of leadership, emotional demands and demands for hiding emotions (Lund et al. 2005).

It is worth underlining that most studies in the literature did not adjust the results for exposure to other work factors, which may explain the higher number of work factors found significantly associated with early exit in these studies, as well as the stronger risk estimates reported for most work exposures.

## Conclusions

The present study indicates that physical and psychosocial work demands—to a stronger extent than lack of resources at work—are risk factors for early exit from the labour market. Examining specific exit routes, work environment factors seem to play a stronger role for the sickness absence route and less pronounced for the unemployment and retirement routes, although the study was underpowered to assess the association of work factors with each exit route in more detail.

Our results indicate that an improvement in working conditions may reduce premature departure from work through different routes, in particular through the reduction of exposure to physical demand and work pace, and, possibly, through an increase of the level of control over working tasks and working time.

## Appendix

See Tables 8, 9, 10.

**Table 8:** Participation at follow-up with non-missing values on all variables by working conditions among 4,201^a^ employees from the baseline interview

Domain	Dimension	*N*	Participated at follow-up and nonmissing on all variables, %	*P*^b^
Physical demands	Walking/standing^c^			0.000
	Low	1793	**60**	
	Medium	858	**56**	
	High	1548	**52**	
	Awkward body postures^c^			0.001
	Low	3406	**57**	
	Medium	370	**54**	
	High	422	**48**	
	Heavy lifting^c^			0.166
	Low	3475	57	
	Medium	368	54	
	High	354	52	
	Repetitive movements^c^			0.183
	Low	2534	57	
	Medium	613	53	
	High	1048	56	
Quantitative demands	Work pace^c^			0.052
	Low	511	52	
	Medium	1061	58	
	High	2622	56	
	Amount of work^c^			0.000
	Low	1497	**54**	
	Medium	1939	**56**	
	High	763	**64**	
Control	Influence at work^c^			0.126
	Low	1659	54	
	Medium	1784	58	
	High	757	56	
	Control over working time^c^			0.005
	Low	1040	**52**	
	Medium	1458	**56**	
	High	1696	**58**	
	Possibilities for development			0.000
	Low	261	**43**	
	Medium	1046	**54**	
	High	2894	**58**	
Relations	Role clarity^c^			0.866
	Medium, low	343	56	
	High	3853	56	
	Quality of leadership^c^			0.103
	Low	678	61	
	Medium	1916	55	
	High	1602	56	

Bold numbers indicate sigificant association to participation

^a^See Fig. 1

^b^P for association of each of the categorical variables to participation at follow-up with non-missing information.

^c^A few employees had missing values at baseline, the least regarding Influence at work (*n* = 1), the most regarding Work pace and Control over working time (*n* = 7).

**Table 9:** Unweighted and weighted associations between baseline work environment dimensions and 134 events 2011/12–2015 of 134 cases early exit from work^a^ among 2351 employees aged 31–60 years in Germany

Domain	Work environment dimension	Model 1 (unweighted^a^). Work environment dimensions not mutually adjusted		Model 2 (weighted^b^). Work environment dimensions not mutually adjusted	
		HR^b^	95% CI	HR	95% CI
Physical demands	Walking, standing	1.02	0.90–1.15	1.02	0.89–1.18
	Awkward body postures	**1.25**	**1.08–1.46**	**1.21**	**1.02–1.44**
	Lifting heavy loads	**1.18**	**1.06–1.31**	**1.23**	**1.03–1.47**
	Repetitive movements	**1.17**	**1.04–1.30**	**1.20**	**1.06–1.36**
Quantitative demands	Work pace	**1.53**	**1.26–1.85**	**1.53**	**1.22–1.93**
	Amount of work	**1.34**	**1.10–1.63**	**1.40**	**1.14–1.72**
Control	Influence at work	**0.80**	**0.67–0.96**	**0.81**	**0.67–0.98**
	Control over working time	**0.82**	**0.70–0.95**	**0.84**	**0.70–0.995**
	Possibilities for development	0.91	0.73–1.14	0.99	0.76–1.29
Relations	Role clarity	1.11	0.80–1.55	1.01	0.71–1.44
	Quality of leadership	**0.82**	**0.69–0.99**	0.83	0.68–1.002

Multiple cox regression, hazard ratios (HR). Bold numbers indicate sigificant HR's

^a^The same results as model 1 shown in Table 4. Shown here again so as to enable direct comparison with the weighted analyses shown to the right in this Table

^b^Weighted so as to control for attrition in the cohort due to gender, age, socioeconomic group, wage level, region, country/city, east/west, educational level and German/non German citizenship (Schiel et al. 2018). Analytical procedure CSCOXREG. The number of weighted exit cases was by coincidence the same as in the unweighted analysis: 134

**Table 10:** Associations between baseline work environment dimensions^a^ treated categorically and 134 events 2011/12–2015 of early exit from work^b^ among 2,351 employees aged 31–60 years in Germany

	*N*	Observed cumulative incidence of early exit, % (n)	*P*^c^	HR^d^	95% CI
Walking/standing			*0.660*		
Low^e^	1074	5 (55)		1	
Medium^f^	478	5 (25)		0.99	0.62–1.77
High^g^	799	7 (54)		1.18	0.79–1.77
Awkward body postures			***0.015***		
Low^e^	1949	5 (105)		1	
Medium^f^	198	6 (11)		1.28	0.68–2.41
High^g^	204	9 (18)		**2.14**	**1.28–3.59**
Heavy lifting			***0.002***		
Low^e^	1970	5 (68)		1	
Medium^f^	197	8 (18)		**1.78**	**1.05–3.05**
High^g^	185	9 (48)		**2.41**	**1.40–4.13**
Repetitive movements			***0.015***		
Low^e^	1438	5 (68)		1	
Medium^f^	323	6 (18)		0.99	0.59–1.67
High^g^	590	8 (48)		**1.70**	**1.17–2.47**
Work pace			***0.002***		
Low^e^	265	3 (9)		1	
Medium^f^	618	4 (27)		**2.41**	**1.11–5.23**
High^g^	1468	7 (98)		**2.31**	**1.64–6.67**
Amount of work			***0.005***		
Low^e^	806	6 (47)		1	
Medium^f^	1057	5 (57)		1.44	0.97–2.16
High^g^	488	6 (30)		**2.22**	**1.37–3.58**
Influence at work			*0.096*		
Low^e^	900	8 (68)		1	
Medium^f^	1030	5 (48)		0.79	0.54–1.17
High^g^	421	4 (18)		**0.56**	**0.33–0.96**
Control over working time			***0.023***		
Low^e^	542	8 (42)		1	
Medium^f^	819	7 (55)		0.72	0.48–1.09
High^g^	990	4 (37)		**0.53**	**0.33–0.83**
Possibilities for development			*0.401*		
Low^e^	111	8 (8)		1	
Medium^f^	564	43 (8)		0.66	0.30–1.43
High^g^	1676	5 (83)		0.59	0.28–1.28
Quality of leadership			***0.012***		
Low^e^	414	8 (35)		1	
Medium^f^	1044	5 (52)		**0.57**	**0.37–0.89**
High^g^	893	5 (47)		**0.54**	**0.34–0.84**

Multiple cox regression, hazard ratios (HR). Bold numbers indicate sigificant HR's

^a^Role clarity was not analysed due to a very skewed distribution (only 8 in the lowest exposed group)

^b^Events of either sickness absence (≥ 18 months or combined with subsequent pension), unemployment (≥ 18 months or combined with subsequent pension) or pension in a 3.6-year follow-up.

^c^This *p*-value denotes—in the cox regression—to what extent each of these categorical variables are associated with early exit from work

^d^Adjusted for age, gender, poverty, fixed-term contract, SEP (4 categories, see Sect. "Materials and methods") and an interaction term for gender and poverty and low SEP. Not adjusted for the other work environment dimensions.

^e^Score below 1.5, reflecting response categories on individual items such as ‘Never’, ‘Never/hardly ever’, ‘Up to a quarter of the time’ or ‘”Seldom”.

^f^Score ≥ 1.5 to  < 2.5, reflecting response categories on individual items such as ‘Up to half of the time’ or ‘Sometimes”.

^g^Score ≥ 2.5, reflecting response categories on individual items, such as ‘Up to three quarters of the time’, ‘More than three-quarters of the time’, ‘Often’ or ‘Always’.
